# Multimodule Web-Based COVID-19 Anxiety and Stress Resilience Training (COAST): Single-Cohort Feasibility Study With First Responders

**DOI:** 10.2196/28055

**Published:** 2021-06-07

**Authors:** Janna Marie Heyen, Noé Weigl, Mario Müller, Stefan Müller, Urs Eberle, Andrei Manoliu, Stefan Vetter, Adam D Brown, Thomas Berger, Birgit Kleim

**Affiliations:** 1 Department of Psychiatry, Psychotherapy and Psychosomatics University of Zurich Zürich Switzerland; 2 Department of Psychology University of Zurich Zürich Switzerland; 3 Department of Psychology University of Bern Bern Switzerland; 4 Zurich Schutz und Rettung Zürich Switzerland; 5 McLean Hospital Belmont Harvard Medical School Boston, MA United States; 6 Department of Psychology New School for Social Research New York, NY United States; 7 Department of Psychiatry New York University New York, NY United States

**Keywords:** anxiety, COVID-19, electronic mental health, feasibility, first responder, mental health, mindfulness, resilience, self-efficacy, sleep quality, stress, training

## Abstract

**Background:**

Since the emergence of COVID-19, health care workers and first responders have been at a high risk for mental health symptoms owing to their exposure to the virus and increased work stress during the pandemic. Although interventions exist to address mental health issues following exposure to disasters, emergencies, and humanitarian crises, considerably less is known about web-based unguided interventions to help mitigate the negative impacts of such events. Additionally, in contexts in which emergencies reduce access to in-person care, remote forms of support are critical, yet there are limited studies on the use of such interventions. Evidence-based, easy-to-use, scalable interventions are direly needed for this population.

**Objective:**

This study aimed to develop and test the feasibility of an unguided electronic mental health program, COVID-19 Anxiety and Stress Resilience Training (COAST), tailored to first responders and health care personnel, based on scientific evidence and empirically based techniques.

**Methods:**

We developed COVID-19–specific training modules focusing on several domains that are previously reported as key to resilience and stress recovery: self-efficacy, mindfulness, sleep quality, and positive thinking. The program was made available to 702 first responders between May and August 2020, during the COVID-19 pandemic. Sociodemographic, work-, and COVID-19–related information was collected, and psychometric questionnaires were completed. We examined user acceptance and user activity, including module choice and participant feedback.

**Results:**

In total, 52 of 702 (7%) first responders to whom we reached out used the program at least once. COAST use was independent of age, sex, or baseline levels of self-efficacy, mindful awareness, sleep quality, and positive thinking (for all, *P*>.39). First responders who had tested positive and those who had been quarantined were more likely to engage in the program. A click count analysis per module showed that participants used the self-efficacy and mindfulness modules most often, with 382 and 122 clicks, respectively, over 15 weeks. Overall, first responders expressed satisfaction with the program.

**Conclusions:**

Engagement of first responders in the multimodule web-based COAST program was feasible and the first responder cohort expressed overall satisfaction with the program. Those in more difficult circumstances, including those in quarantine and those who tested positive, may be more likely to engage in such programs. Further controlled studies could pave the way for efficacy studies and the development of additional modules, including just-in-time interventions or blended interventions combining individual use of an unguided self-help intervention, such as COAST, with subsequent individual psychotherapy for those who continue to experience stress and psychological symptoms.

## Introduction

Since early 2020, COVID-19 began to severely impact the daily lives of a majority of the world’s population after a cluster of infections was first identified in China as early as December 2019 [1]. In most countries, including Switzerland, severe measures were taken to prevent the further spread of SARS-CoV-2, including strict safety and hygiene rules and physical distancing. Lockdown and physical distancing measures, along with perceived unpredictability and uncertainty, may lead to social isolation, loss of income, loneliness, inactivity, limited access to basic services, and other compromises. COVID-19 has thus resulted in an increase in known risk factors for psychiatric disorders such as depression and anxiety [2]. A number of studies documented significant effects on the population’s mental health, with studies reporting higher levels of depression in the general population [3]. Health care workers and first responders were often found to be at high risk for mental health issues owing to increased exposure to the virus and higher work stress during the pandemic [4-7], and they experience increased mental health symptoms [8].

With physical distancing and other restrictions in place and a decreased mental health system capacity, calls for increased investment in telehealth and telepsychiatry, as well as other digital mental health solutions, have emerged [9-11]. Digital mental health prevention and intervention programs offer effective, often unguided, and scalable solutions to improve mental health. These can often be used anonymously, making their use lower threshold than that of in-person therapy [12-14], and are acceptable by users [15]. Early interventions could be key to prevent overburdening mental health care systems during and after the COVID-19 pandemic [16,17]. Indeed, some electronic mental health (e-mental health) interventions have already been implemented in different countries during the pandemic [18-20]. Studies on the feasibility and effectiveness of e-mental health interventions for medical personnel and first responders, however, remain scarce. A recent review reported 3 studies on mental health programs specifically targeted at medical personnel during the pandemic through e-mental health approaches [21]. However, there is evidence for the efficacy of e-mental health approaches for these populations in other circumstances. A blended e-mental health intervention to manage weight was effective in a sample of first responders and included interventions on sleep and psychological symptoms [22]. Another 3-month web-based intervention to reduce work-related fatigue and improve work function resulted in a small positive effect on overall stress in health care workers [23]. E-mental health interventions are thus a promising way to support health care workers and first responders in general and possibly during crises, including the current COVID-19 pandemic. Not all current approaches are based on empirical evidence or employ principles of evidence-based treatment approaches for psychiatric disorders, such as cognitive behavioral therapy [24]. To help bridge this gap, we developed a research-based e-mental health program tailored to first responders and health care personnel, which is based on robust scientific evidence and empirically based techniques, is self-paced and unguided, and can be delivered anonymously. Our study aimed to (1) develop research-based, web-based, COVID-19–specific training modules focusing on the key areas of self-efficacy, mindfulness, sleep quality, and positive thinking to increase resilience in health care workers and first responders; (2) assess sociodemographic, COVID-19–, and stress-related characteristics of first responders opting into the program; and (3) examine user acceptance and activity, including their choice of different modules and feedback.

## Methods

### Study Design

This study was designed as single-cohort feasibility study to evaluate a COVID-19 Anxiety and Stress Resilience Training (COAST) program and receive user feedback for future adaptation of our modules. The study was approved by the ethics committee of the philosophical faculty of the University of Zurich. We included first responders who were offered free and unlimited access to the program. The first users and study participants joined COAST in May 2020, and final questionnaires were completed in July 2020. Our analyses encompass an overall period of 15 weeks, during which participants were followed-up during their individual use of the program.

### The COAST Program

COAST comprises 4 intervention modules to increase resilience to stress due to the COVID-19 pandemic. These modules were made available on a website for free and self-paced perusal by our participants. The self-paced option appeared relevant to adapt to the busy schedule of health care workers during the pandemic. All 4 modules are standalone modules, targeting (1) self-efficacy, (2) sleep quality, (3) mindfulness, and (4) gratitude and positive reframing. The choice of these module topics was based on previous reports that identified them as useful targets to improve resilience to stress and adversity [25-28] and on the target population’s documented preference for e-mental health interventions to be focused on well-being rather than on ill health [29].

Each module contained explanations and mini-interventions that users could engage with in their daily lives, based on previous results and protocols and adapted for web-based use. The self-efficacy module was based on the findings that activating autobiographic memories of perceived self-efficacy can help strengthen clinically relevant factors for tolerating distress [30] and promote relevant cognitive processes and problem-solving that might help patients recover from stress [31] [Multimedia Appendix 1]. Users are asked to recall 3 memories of situations that they handled well and write down which hurdles they overcame and which traits, qualities, and strengths helped them overcome these hurdles. The sleep module consists of a worry diary and tips for better sleep hygiene, both proposed by Altena et al [32] and the European Academy for Cognitive-Behavioral Therapy for Insomnia. The module on mindfulness includes various audio files with guided meditations. Studies on populations of health care workers found mindfulness to be associated with reduced depressive symptoms, more adaptive defense mechanisms against stress, lower burnout and stress levels, and higher life satisfaction [33-35]. These findings are supported by a meta-analysis that reported mindfulness-based interventions to be an effective tool to help medical personnel cope with stress [26]. COAST’s gratitude module involves a gratitude diary, which users can fill in daily. Killen and Macaskill [36] reported that a gratitude diary could positively influence well-being and found no difference in efficacy between the paper-based and web-based versions of this intervention. The use of interventions that train gratitude has further been proven effective in various studies [36-38], partially focusing on health care workers.

Decisions in favor of shorter interventions and questionnaires were taken considering previous reports highlighting the occasional low participation rates of health care workers and first responders and their high workload [39].

### Recruitment

We recruited first responders from Schutz & Rettung Zürich, an urban first responder organization that provides first aid services in the Greater Zurich Region. For our study, all 702 first responders in the service, mostly including ambulance workers and fire fighters, were invited to use COAST via email. In total, 52 of them used COAST at least once and were included in the study. The emails contained an invitation to take part in the study, with the purpose of evaluating COAST, and a link to the first set of study questionnaires (baseline) immediately after registration. Furthermore, users were asked to fill in a short questionnaire on the website and were sent reminders to do so. Inclusion criteria were being a first responder at the abovementioned institution, providing consent to participate in the study, and signing up for the COAST program. Participants in the study were offered to enter a raffle and win 1 of multiple vouchers by entering a draw at the end of each external questionnaire. Owing to the exploratory nature of the study and our interest in feasibility indices that are largely independent of sample size, power analysis was not conducted.

### Procedure

Participants chose an anonymous username, as the privacy of e-mental health interventions had been previously raised as an important issue pertaining first-responder interventions [22]. They were also asked to complete self-report questionnaires that were sent via email and could be completed on the internet. These links were sent at three timepoints: at baseline (ie, after registration and prior to starting COAST) (questionnaire 1), at 2 weeks (questionnaire 2), and at 4 weeks (questionnaire 3). Questionnaires 2 and 3 also included a user experience questionnaire. They also were asked to complete several items covering each module’s target outcome (self-efficacy, mindfulness, sleep, and optimism) on the COAST website.

First responder personnel in Zurich, who were interested in engaging with the COAST program, were sent a log-in code for the program via email. The program was delivered via a custom-built university website. For those participating in the study, the code was also used to match program-based data (including user indices and questionnaire items on the website) from the COAST website, with other questionnaires that were sent to participants via a link to their browser, and completed on the internet to ensure anonymity. The users agreed to the use of their data for research purposes when entering their code prior to their engagement with the COAST program and at the beginning of each questionnaire that was filled in outside of the program. All questionnaires were filled in on the internet. A total of 3 reminder emails were sent for questionnaire completion.

### Measures

Participants filled in items embedded in the COAST program as well as separate questionnaires at baseline and at 2 and 4 weeks’ follow-up. Questionnaire completion was not mandatory for participating in the program, and the number of users who completed questionnaires varied. We thus report on different subsamples.

We used standardized, validated, self-reported measures at baseline (questionnaire 1) and follow-up (questionnaires 2 and 3), including the Perceived Stress Scale [40] (Cronbach *α*=.95), the 9-item Patient Health Questionnaire [41] (Cronbach *α*=.83), the 7-item Generalized Anxiety Disorder Scale [42] (Cronbach *α*=.89), the General Self-Efficacy Scale [43] (Cronbach *α*=.76), and the Posttraumatic Stress Disorder Checklist of the Diagnostic and Statistical Manual of Mental Disorders (5th edition) [44] (Cronbach *α*=.95). An adapted version of the 8-item Client Satisfaction Questionnaire (CSQ-8) [45] was used in questionnaires 2 and 3 to assess the users’ satisfaction with the web-based program. For our study, we adapted CSQ-8 items to refer to the web-based program rather than the service, the clinic, or the treatment. No further modifications were made. In our sample, the measure had a high internal consistency (Cronbach *α*=.94). We also employed an open question on potential further suggestions to optimize the program (eg, “Do you have ideas, suggestions, or criticism for us that could help us make COAST better?”).

Within the COAST program, sociodemographic, work-, and COVID-19-related questions were completed by the participants. We also included key questions corresponding to each of the target modules obtained from the validated questionnaires. Users were asked to fill in the questions when they started using the program. The questions measured stress (“How nervous or stressed do you feel today?”), perceived self-efficacy (“How much do you currently believe in being able to change things in your life?”), mindfulness (“I am in contact with my experiences here and now”), sleep quality (“How would you judge your sleep quality since the last log-in?”), and optimism or positive thinking (“I have a positive outlook on the future”). Users answered the questions on a scale of 0-10 for stress, self-efficacy, and optimism and 0-3 for sleep quality and mindfulness.

Activity scores were calculated over 15 weeks on the basis of the users’ activity in the program, reflecting the number of times individual pages of the program were accessed, including repeated access to the same page.

### Statistical Analysis

All statistical analyses were conducted in RStudio (version 1.3.959) for Mac OS [46]. We calculated mean (SD) and percentage values for sociodemographic and COVID-19–related variables and for other questionnaire and activity scores. The number of clicks per page was grouped per module, and clicks per module were counted to determine the differences in user activity (n=52). Correlations were calculated among questionnaire item scores and user activity. Pearson correlation analysis was used where normality distribution assumptions were met and adjusted to nonparametric testing in all other cases. We applied 1-sided significance testing and a *P* value of .05 to indicate significance.

## Results

### Characteristics of First Responders Opting Into the COAST Program and Their Association With User Activity

Sociodemographic information and scores for in-program questions are displayed in Table 1. The sample comprised 52 first responders, of which 42 (42.9% female) completed in-program questionnaires. The mean age was 43 (SD 10.53) years, with 14 (26%) participants having been quarantined at some time point, and 1 (2%) was quarantined at the time of filling in the questionnaire. Moreover, 21% worked or had worked in direct contact with patients with COVID-19. In total, 1 (2%) participant had tested positive for COVID-19, and 6 (24%) stated that they belonged to a COVID-19 risk group for severe disease progression. Single questions within COAST yielded low perceived stress and sleep problems in our sample while showing high self-efficacy and optimism, as well as an intermediate level of mindfulness.

**Table 1:** Descriptive statistics (N=42).

Characteristic		Value
**Sex, n (%)**		
	Female	18 (42.90)
	Male	24 (57.10)
Age (years), mean (SD)		43.79 (10.53)
**Quarantine, n (%)**		
	At any time	11 (26.19)
	At time of study	1 (2.38)
Direct contact with patients with COVID-19, n (%)		9 (21.43)
Member of a risk group, n (%)		10 (23.81)
Tested positive for COVID-19, n (%)		1 (2.38)
Activity score, mean (SD)		15.46 (10.31)
**Question scores, mean (SD)^a^**		
	Perceived Stress	3.39 (2.83)
	Perceived Self-Efficacy	6.85 (2.27)
	Sleep	1.16 (0.85)
	Mindfulness	1.63 (0.97)
	Optimism	7.23 (2.20)

^a^Questionnaires were based on single items completed within the program while working on COAST modules. Scores ranged 0-10 for stress, self-efficacy, and optimism and 0-3 for sleep quality and mindfulness, with higher scores indicating more perceived stress, self-efficacy, lower sleep quality, and greater mindful awareness.

COAST participants were representative in terms of age and sex, for the overall population of first responders from the aforementioned first responder organization, one of the largest urban first responder units in Switzerland, and there were no significant differences between the 2 populations in these variables. However, the proportion of positive COVID-19 tests was higher among COAST participants than among the total sample of 702 first responders in the organization (2.4% vs 0.7%, respectively), and 26% of COAST participants reported having been in quarantine at some time, while this number was at less than 1% in the overall first responder population.

### Participant Acceptance, Activity, and Feedback

We found no significant correlations between user activity (ie, number of clicks) and sociodemographic, COVID-19–, or work-related items (ie, previous COVID-19 infection, age, sex, current quarantine, etc). Perceived stress and self-reported scores relating to module targets (self-efficacy, mindfulness, sleep quality, and optimism) were also unrelated to user activity scores (Table 2).

Analysis of the click count per module indicated that the self-efficacy module was used the most (amounting to 382 clicks), followed by the mindfulness (122 clicks), sleep (103 clicks), and gratitude (47 clicks) modules. The mean activity score of all users was 15 (SD 11.11) clicks in the program, assessed over a period of 15 weeks. Activity scores ranged 0-54 clicks, while the time spent on the pages was not measured. We detected 3 outliers through visual analysis of the boxplot and excluded them from the activity scores.

On average, users were satisfied with the program, as indicated by a mean score of 21.42 (SD 4.08; range 8-32) on the CSQ-8, representing “mild satisfaction.” Some participants reported difficulties using a web-based format or wanted more visualization of the content. One user was concerned about confidentiality, specifically with regard to work-related answers.

**Table 2:** Association between program activity levels (ie, click count score) and sociodemographic variables, perceived stress, and scores mapping on module targets.

Variable	Association with program activity^a,b,c,d^	*P* value
Age	0.03^c^	.57
Sex	0.02^a^	.56
Quarantine (at any time)	0.02^a^	.44
Direct contact with patients with COVID-19	0.13^a^	.21
Member of a risk group	0.05^a^	.38
Perceived stress	–0.18^b^	.81
Perceived self-efficacy	–0.06^b^	.39
Sleep quality	–0.05^a^	.60
Mindfulness	0.05^a^	.40
Optimism	–0.03^b^	.55

^a^Point biserial correlation coefficients.

^b^Spearman correlation coefficients.

^c^Pearson correlation coefficients.

^d^Correlations with positive COVID-19 test results could not be determined because only 1 person in the group that responded to the respective questionnaires had received a positive test result.

## Discussion

### Principal Findings

We developed an evidence-based, multimodule, web-based COAST program to increase resilience to stress among health care workers and first responders during the COVID-19 pandemic. Here we report data from a feasibility study. COAST was actively used by 52 first responders, which accounts for 7% of the overall first responder group targeted and invited to participate during the COVID-19 pandemic. COAST use was independent of age, sex, and perceived stress, as well as scores mapping on the content of the module target of the respective intervention (including self-efficacy, mindfulness, sleep quality, and optimism). First responders who had tested positive for COVID-19 and those who had been quarantined were more likely to engage with the program. This suggests that we indeed may have reached those who are more and directly affected by the COVID-19 pandemic and are potentially in need of an intervention; however, a decreased workload during quarantine may also have contributed to this. Individual users mostly engaged in the self-efficacy memory module, followed by the mindfulness, sleep quality, and gratitude modules. Overall, first responders expressed satisfaction with the program.

The use of web-based technology is gaining popularity in mental health care, and these technologies have become increasingly available and affordable, thus lending themselves to implementation in the current first responder setting. Owing to their busy and challenging work environment, we expected to face challenges with regard to the engagement of this population with our program. Previous studies have indeed confirmed that first responders [29] and medical personnel working at a hospital [39] may have very specific needs as well as expectations from interventions. Hence, we developed COAST to be easily accessible and self-paced and to fit to individual needs. Indeed, we found large interindividual variability in COAST participation, with the number of clicks and activity low among some but regular and high among other first responders. Interestingly, activity was independent of self-reported self-efficacy, mindfulness, sleep quality, optimism, and perceived stress. As expected, some modules were used more than others. The self-efficacy memory module was used most often. Self-efficacy may emerge as a key construct underlying risk and resilience in relation to COVID-19, given the unpredictability of the COVID-19 pandemic and lack of controllability and agency experienced by some individuals. Theoretical models [25] and numerous studies with trauma-exposed individuals have found that self-efficacy is an important mechanism underlying risk and recovery among first responders and emergency personnel [47-49]. Participants in this study may have been drawn to this module in an attempt to improve self-regulation and to experience a sense of control by recalling previous challenges and obstacles that had been overcome. Although these data do not include self-efficacy–related outcomes, interest and engagement in this module may offer a promising strategy for reducing distress and maintaining well-being, as studies have shown that perceptions of self-efficacy can be increased among healthy [50,51] and clinical populations [30,31]. Higher levels of self-efficacy have been associated with greater problem-solving abilities [51] and a higher level of persistence, as well as changes in the activity of brain regions linked to emotional regulation [52]. By instructing and supporting first responders’ recall of autobiographical self-efficacy memories, such adaptive phenomena may have been activated, and self-efficacy may have been increased. While these studies support these assumptions, processes in the field, such as those investigated here among the first responder population, will have to be investigated further to elucidate the precise mechanisms of action of individual modules, such as the self-efficacy module.

The second-most frequently used module was mindfulness. This is in line with recent efforts and success of implementing mindfulness interventions for mental health and other health workers [53,54]. Previous studies have reported significant effects of mindfulness programs in scalable, practical ways, including successful delivery of web-based mindfulness training in high-risk workplace settings and first responders in an entirely web-based version, as in our study [50]. Again, we can only speculate the mechanisms of action of this training program. In a previous study on frontline medical workers, we found that lower activation of the arousal system, indexed by activation of the arousal system during an emotion regulation task, was associated with increased resilience during subsequent stressful medical work [55]. Since one demonstrated effect of mindfulness is a change in gray matter concentration in several brain regions, including the locus coeruleus arousal system [56], such changes may well underlie the effects of mindfulness interventions and could be important to the first responder populations. In other words, successful engagement in mindfulness may lead to changes in brain regions that contribute to a more adaptive arousal system and enhanced well-being after mindfulness practice. However, we might have hastened to draw further conclusions about the precise effects of our modules. The same applies to ranking activity in the modules. In addition to personal choice informed by module content, module choice may also reflect the position of the module on the web layout of our program, rather than reflecting the preferences of individual users.

Only a subgroup of participants provided feedback through questionnaires, however this could potentially be of use for further program development. Among the points raised by the participants were privacy concerns, which is commonly reported in such populations [22,29] and should be included in future developments. Specifically, users were concerned about information they provided and that might be fed back to line managers and employers. This was not the case in our program and should be approached similarly by future programs. Our and other future programs should also consider user preferences in terms of graphics and design and should increase and optimize graphical module features, which were rather basic in our program and could be enriched and animated in several ways and include elements of personalization; that is, users would create their very own character that accompanies them, guides them through the modules, and potentially encourages individual engagement.

Our sample was representative of the targeted population of first responders, but the health care system in Switzerland may not be comparable with those in other countries; hence, our findings may not be generalizable to other health care professionals during the COVID-19 pandemic. A country’s economy and socioeconomic differences and the severity of the outbreak in the country have been shown to influence the impact of the COVID-19 pandemic on a population [17,18,57]. All our study participants were employed as of this writing, thus also limiting the generalizability of our findings to health care workers and first responders from low-income countries or those with lower job stability.

### Limitations

Our study is not without its limitations. First, while we noted significant interest in COAST among our study participants and subsequent activity in the program among those who registered, the response rate to the external questionnaires (questionnaires 1-3), which we administered to our participants for program evaluation, was low and these could therefore not be evaluated. Future programs should make module use conditional on completion of a core set of questionnaire items. Second, all questions were self-reported questionnaire items and were thus associated with known challenges, including a retrospective response bias, social desirability [58], and being affected by current mood states [59]. Further, the scores reported here have been obtained from single questions on each subject, and although these were based on full-length questionnaires, their separate use is not validated. Third, the exact timing of in-program questions and their relation to user progress in COAST could not be determined. We also did not obtain exact results on how long users spent in a given module. Our results are thus preliminary and future studies will need to assess larger samples and test for controlled effects of the program’s effectiveness, which could then help establish the program’s effectiveness in decreasing the symptoms of stress and change scores in our target processes for self-efficacy, mindfulness, sleep quality, and positive thinking. Finally, and perhaps most importantly, the response rate of the overall population of first responders was 7% and thus rather low. Such challenges to engage health care workers (and indeed other groups) in e-mental health interventions during the COVID-19 pandemic have been well documented. Chen et al [39] reported the reluctance of hospital staff in China to use available support. They adjusted their program to include staff feedback and established resting rooms and provided in-person counseling services. Ketelaar et al [23] suggest using blended care to reduce attrition and boost participation, and this option should be considered for the current program. Another study from Wuhan [37] reported that difficulties with engagement might stem from issues with reduced trust and a heightened sense of stigma, as well as a high workload, and implemented anonymous interventions and daily reminder messages. Additional suggested challenges and reasons for low participation could be low perceived need, technical problems, and unattractive channeling toward their intervention [23]. While we are not aware of any technical problems in the COAST program, the low perceived need could have contributed to low participation, as our baseline questionnaire indicated low mental health symptoms among most respondents. In the future, stepwise approaches could be implemented, including screening for at-risk participants, who will then be offered further interventions rather than a one-size-fits-all approach. Privacy concerns seem to be key in the target population [22,29], and participants often requested that a web-based intervention should be independent from their employer. While COAST was in fact developed, run, and analyzed entirely independently from the employer, the program and study was announced and offered to the participants through their institutional email.

### Conclusions

Despite these limitations, our study has practical and clinical implications for prevention and intervention science. We reached a small, albeit significant subgroup of first responders who actively used the program and provided feedback. Advantages of such e-mental health interventions include their application and use without physical contact and their scalability, as we can reach more clients than would be possible face-to-face. These are critical features during crises, such as the current pandemic. More controlled studies on developing and adapting web-based interventions tailored to the preferences and needs of health care workers and first responders during the COVID-19 pandemic are needed. Such studies could also pave the way for additional novel interventions, such as just-in-time interventions or blended interventions combining individual use of a self-paced intervention with subsequent individual psychotherapy for those who continue to experience stress and psychological symptoms.

## Appendix

Multimedia Appendix 1 Exemplary screenshot: COAST Self-efficacy module.

## Abbreviations

COAST: COVID-19 Anxiety and Stress Resilience Training
CSQ-8: 8-item Client Satisfaction Questionnaire
e-mental health: electronic mental health
