# Supplementary material for: Multimodule Web-Based COVID-19 Anxiety and Stress Resilience Training (COAST): Single-Cohort Feasibility Study With First Responders
Source: JMIR Form Res. 2021 Jun 7;5(6):e28055. doi: 10.2196/28055 (PMC8189283; doi:10.2196/28055)
Supplement: Multimedia Appendix 1 [file formative_v5i6e28055_app1.doc]

#### **Multimedia Appendix 1:** Exemplary screenshot: COAST Self-efficacy module.

####


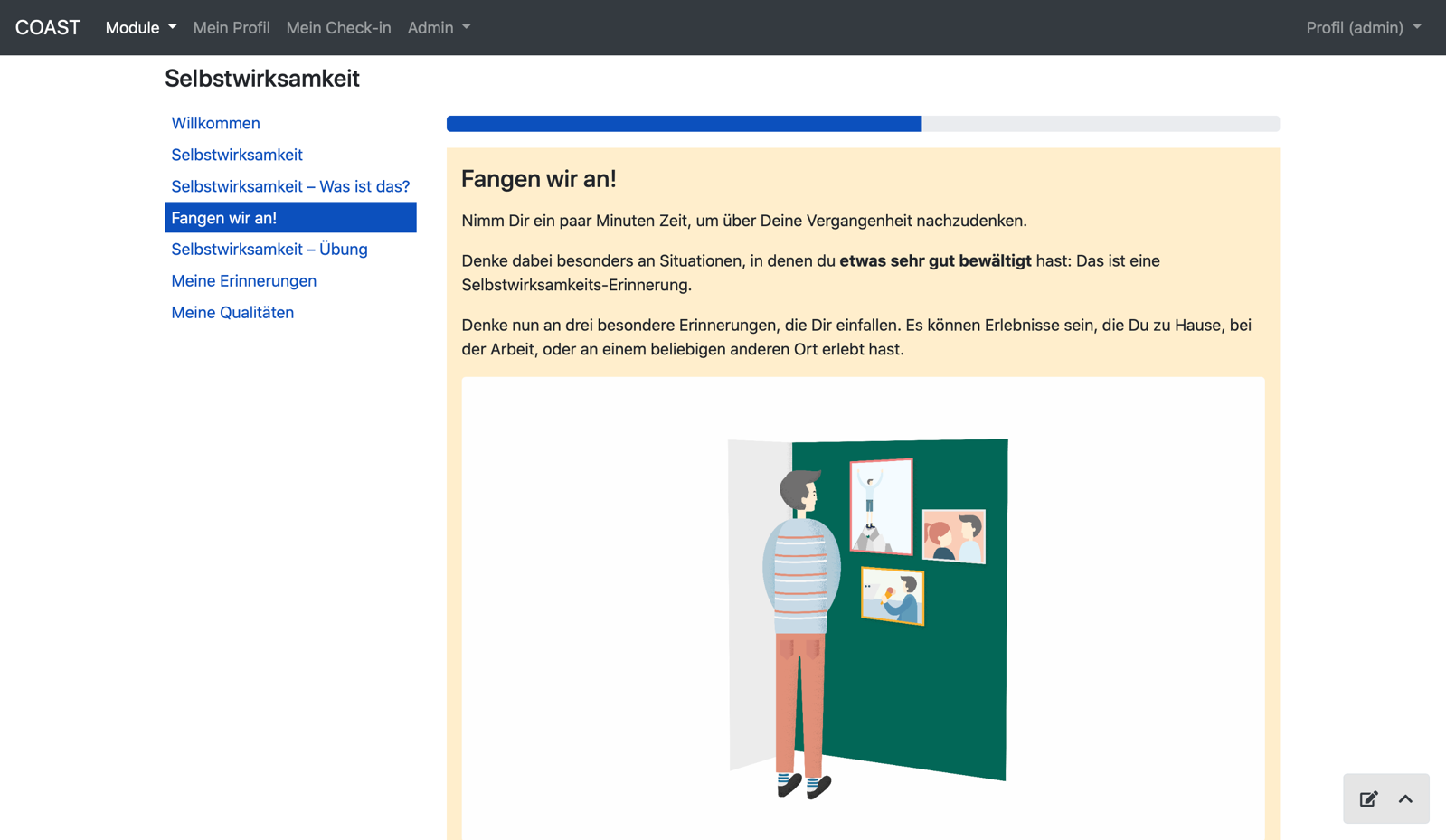


Note: The screenshot depicts the intervention participants are invited to engage in in the self-efficacy module. Following a general introduction into the self-efficacy construct, users are prompted to retrieve three self-efficacy autobiographical memories. They are then guided vividly imagine these personal memories and extract personal strengths and qualities that helped them handle the situation well. Participants can use text fields on the website that are later available for review. All modules follow a similar structure, i.e., general introduction, engagement in an intervention with possibilities to make notes or listen to an audio file (for mindfulness module) and wrap up and encouragement to continue practicing the exercise.
